# Evaluating the Effectiveness of External Molecular Proficiency Testing in the Global Polio Laboratory Network, 2021–2022

**DOI:** 10.3390/pathogens13111014

**Published:** 2024-11-19

**Authors:** Nancy Gerloff, Cara C. Burns

**Affiliations:** Division of Viral Diseases, Centers for Disease Control and Prevention (CDC), Atlanta, GA 30329, USA

**Keywords:** poliovirus, molecular external quality assessment, nucleic acid testing, World Health Organization, Global Polio Eradication Initiative, quality assurance program, accreditation, proficiency panel testing

## Abstract

In the Global Poliovirus Laboratory Network (GPLN), participation and successful completion in annual proficiency test (PT) panels has been a part of the WHO accreditation process for decades. The PT panel is a molecular external quality assessment (mEQA) that evaluates laboratory preparedness, technical proficiency, the accuracy of data interpretation, and result reporting. Using the Intratypic Differentiation (ITD) real-time RT-PCR kits from CDC, laboratories run screening assays and report results in accordance with the ITD algorithm to identify and type polioviruses. The mEQA panels consisted of 10 blinded, non-infectious lyophilized RNA transcripts, including programmatically relevant viruses and targets contained in the real-time PCR assays. Sample identities included wildtype, vaccine-derived (VDPV), Sabin-like polioviruses, enterovirus, and negatives, as well as categories of invalid and indeterminate. The performance of individual laboratories was assessed based on the laboratory’s ability to correctly detect and characterize the serotype/genotype identities of each sample. The scoring scheme assessed the laboratory readiness following GPLN guidelines. Laboratories receiving mEQA scores of 90 or higher passed the assessment, scores of less than 90 failed and required remedial actions and re-evaluation. In 2021 and 2022, 123 and 129 GPLN laboratories were invited to request the annual PT panel, and 118 and 127 laboratories submitted results, respectively. The overall results were good, with 86% and 91.5% of laboratories passing the PT panel on their first attempt in 2021 and 2022, respectively. Most labs scored the highest score of 100, and less than one quarter scored between 90 and 95. Less than 10% of submitting laboratories failed the PT, resulting in in-depth troubleshooting to identify root causes and remediations. Most of these laboratories were issued a second PT panel for repeat testing, and almost all laboratories passed the repeat PT panel. The results of the 2021 and 2022 annual mEQA PTs showed that, despite the COVID-19 pandemic, the performance remained high in the GPLN, with most labs achieving the highest score. For these labs, the real-time PCR assay updates that were implemented during 2021–2022 were carried out with full adherence to procedures and algorithms. Even initially failing labs achieved passing scores after remediation.

## 1. Introduction

The Global Polio Laboratory Network (GPLN) has been the pivotal instrument for aligning poliovirus diagnostic methodologies across the global landscape of technologies. The global surveillance for acute flaccid paralysis (AFP) cases tests more than 200,000 specimens during a year [1]. The testing follows a specific algorithm using virus isolation in susceptible cell lines followed by real-time reverse transcription PCR (RT-PCR) testing and sequencing if required. Most labs perform one of the three main workstreams or a combination of them. Over the last decades, the GPLN has grown from a handful of laboratories to 144 GPLN laboratories in 2024. The accreditation process includes demonstrated proficiency in the four-tiered format for each of the tiers: (I) virus isolation for polioviruses (PV), (II) intratypic differentiation of polioviruses (real-time RT-PCR), and (III) sequencing, as well as successful completion of an on-site inspection.

**Figure 1:**
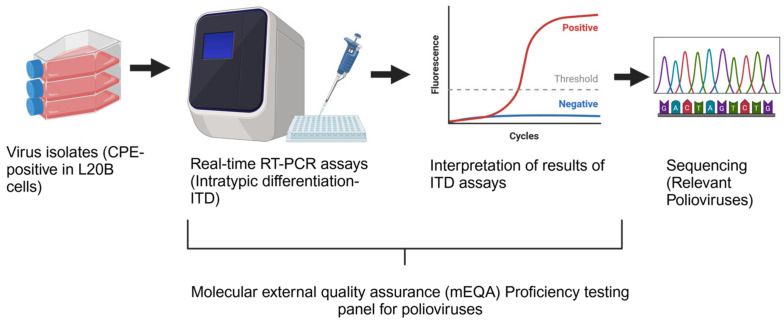
Scheme of annual poliovirus proficiency testing workflow for the molecular external quality assurance (mEQA) using real-time RT-PCR (ITD testing). Separate PT panels exist for virus isolation and sequencing components of poliovirus testing.

In the GPLN, laboratories have participated in the annual proficiency test (PT) panel as a required component of the WHO accreditation process [2]. The molecular external quality assessment (mEQA) or molecular proficiency testing (PT) panel evaluates the ability to type poliovirus isolates in conjunction with laboratory preparedness, technical proficiency, the accuracy of data interpretation, and result reporting (Figure 1). Using the Intratypic Differentiation (ITD) primer and probes from CDC, laboratories run a suite of real-time RT-PCR screening assays and report results in accordance with the ITD algorithm to identify and type polioviruses. It is now used by GPLN laboratories in all six WHO regions.

As one of the Global Specialized Laboratories, the Polio and Picornavirus Branch at the CDC in the United States has been instrumental in assay and method development, technical support, reagent provision, and the administration of the molecular proficiency testing panels. In early surveillance, laboratories performed serotyping by microneutralization using cross-adsorbed antibodies. After serotyping, ITD allowed differentiation between wild and vaccine strains [3]. Starting in 2004, the laboratory performance for methods such as probe hybridization, or ITD by ELISA, or gel-based RT-PCR, which have been replaced by a suite of real-time reverse transcription PCR (RT-PCR) assays in 2009, abbreviated as ITD [4,5,6]. Since 2013, the suite of real-time RT-PCR assays has undergone several iterations (version 1.0 to version 4.0) to adjust the assays for changing virus circulation patterns and vaccine usage (i.e., wildtype PV 1 genetic drift, cessation of oral polio vaccine type 2 in 2016, and the usage of novel OPV2 in 2020) [7,8,9]. Released in 2023, the ITD version 6.0 consists of assays with 11 targets that are tested for polioviruses [10].

Poliovirus diagnostic testing starts by inoculating acute flaccid paralysis (AFP) stool suspensions into susceptible cell lines, rhabdomyosarcoma (RD), and L20B cells (murine recombinant cells with the poliovirus receptor), followed by passaging, which is termed virus isolation. If the cytopathic effect (CPE) is observed in cells, isolates will be directly used in real-time RT-PCR without an RNA extraction step. A separate virus isolation proficiency testing panel is distributed by the Global Specialized Laboratory RIVM (The Netherlands) and aims at testing proficiency in isolation of poliovirus, cell sensitivity as well as the ability to determine CPE positivity [11]. Isolates positive in the virus isolation are tested in PCR (ITD), followed by reflex assays (VDPV assays to confirm Sabin 1 or Sabin 3). Any programmatically relevant serotypes and genotypes must be sequenced for confirmation (wildtype 1, any PV type 2, wildtype 3, and vaccine-derived polioviruses, VDPV). Oral polio vaccine strains, such as Sabin-like 1 and Sabin-like-3, do not require sequence confirmation. The ITD result is used to select appropriate sequencing primers for amplification of the complete Viral Protein 1 (VP1). The resulting Sanger sequence can be genotyped and classified as wild or vaccine-derived poliovirus [10,12].

## 2. Methods

### 2.1. Participating Laboratories and Preparation of the mEQA Panel

In 2021 and 2022, accredited and new GPLN laboratories from all six WHO regions were invited to participate in the annual proficiency testing panel. The PT panels were made available to International Reagent Resource (IRR) registrants and GPLN member labs between September 2021 and November 2021 and October 2022 and December 2022. Laboratories participating in the mEQA use the suite of ITD assays to discriminate among serotypes 1, 2, and 3, as well as distinguish wildtype viruses from vaccine-related polioviruses (Figure 1). There are two reflex real-time RT-PCR assays that are used as a screen to flag viruses that have mutated at key target sites (VDPV1 and VDPV3 assays). Use of the correct reporting scheme and correct interpretation of the assay results are crucial steps to pass the PT panel. Participating laboratories were provided with a standardized reporting sheet that collected test results and testing information, such as equipment used for testing (PCR cycler) and reagent information (lot numbers, expiration dates). In 2021–2022, the following real-time RT-PCR cyclers were validated for ITD and VDPV assays and GPLN use (AB 7500, AB 7500 Fast, QuantStudio 5 [Thermo Fisher: Applied Biosystems, Foster City, CA, USA]; CFX96 [Bio-Rad, Hercules, CA, USA]; Rotor-Gene Q [Qiagen, Germantown, PA, USA]; MxP3005X [Agilent, Santa Clara, CA, USA]).

CDC (Atlanta, GA, USA) and the IRR (Manassas, VA, USA) are the manufacturers of the PT panels. Whereas CDC designs and determines suitability in the quality assessment and quality control (QA/QC) process, IRR is responsible for aliquoting, lyophilizing, labeling, and packaging the mEQA panels for distribution in the GPLN. The mEQA panels were five versions, with each containing a randomized set of 10 blinded non-infectious RNA transcripts to test the ability to detect and type the nucleic acid of poliovirus types 1, 2, and 3 or mixtures thereof. The 10 RNAs also contained non-poliovirus transcripts made up only of Enterovirus RNA (5′ Untranslated region, 5′-UTR), Qβ bacteriophage RNA transcript, or negative template control (water). Each version was composed of multiple RNA transcripts (in total, 16 different RNA transcripts were used, for a total of 30 PT panels per version, Table 1). The mEQA addresses levels of sensitivity on different thermocyclers and assays sensitivity differences (i.e., redundant assays are positive for multiple targets for one RNA transcript). Triplicates of each of the dispensed and lyophilized RNA transcripts were tested in each of the 6 ITD assays (for ITD version 5.2 only, 5 assays were run, plus the nOPV2 Supplemental assay) and the respective VDPV assays (Sabin 1 or Sabin 3-positive transcripts only). The PT panel was evaluated on the GPLN cyclers, including AB 7500, CFX96, and Rotor Gene Q.

PT panel version numbers are omitted from the ordering catalog and box label, and the RNA transcripts are anonymized as Unknown A through Unknown J. The version of the PT panel is tracked by the CDC; version identities are not disclosed to any participating laboratory prior to testing. Labs were instructed to use either the ITD version 5.2 (or version 5.1), the Supplemental nOPV2 assay (not required for all labs), and the VPDV assay kit version 5.2 kit (or non-expired version VPDV 5.0) for testing in 2021, and 2022.

### 2.2. Data Collection and Analysis

PT panel results were submitted using a website submission tool starting in the 2022 season. In 2021, PT panel results were evaluated after submission either through email or the GPLN management submission database, including raw data files, results following the WHO GPLN reporting scheme, and completed reporting sheets (standardized documents). Evaluation of raw data files was performed by a subject matter expert in the CDC Polio Team, and PT panel scores and comments were communicated via email or the submission tool in a standardized format. Each lab received detailed feedback about deductions, and the errors were categorized for annual tracking. Only one submission per lab was accepted for an annual score. New labs were assessed on a practice PT panel first before being invited to participate in the annual PT panel assessment and submission to the website in 2022.

## 3. Results

### 3.1. The GPLN Landscape and Overall Proficiency

The performance of individual GPLN laboratories was assessed based on the laboratory’s ability to correctly detect and characterize poliovirus serotype and genotype identities deemed programmatically critical to the Global Polio Program. The scoring scheme assesses the laboratory readiness following GPLN guidelines based on relevant serotypes, interpretation, and the reporting algorithm used between 2021 and 2022 (Table 2). Laboratories receiving mEQA scores of 90 or greater passed the annual assessment; scores of less than 90 failed and required remedial actions and re-evaluation, including repetition of another PT panel.

**Figure 2:**
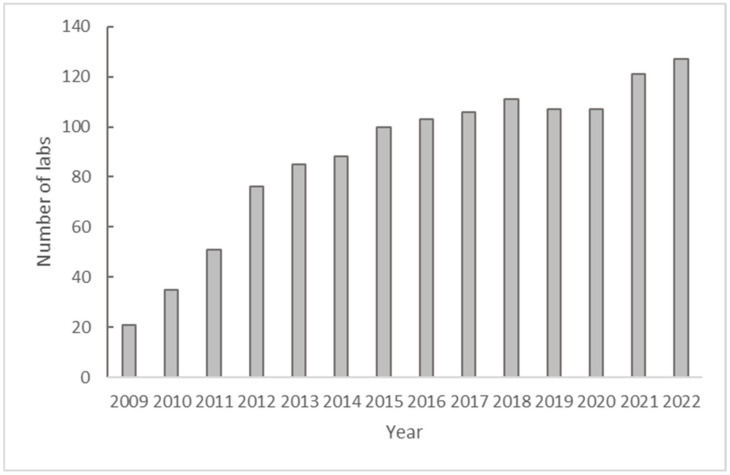
Number of GPLN laboratories participating in the annual mEQA PT panel between 2009 and 2022 using real-time RT-PCR (ITD testing).

In 2021, 123 laboratories were invited to request the annual PT panel, with five labs not participating or not submitting results by the close of the PT panel season (Figure 2). The overall results were good, with 105/123 (86%) of participants passing the PT panel on their first attempt (Table 3). About a quarter of the laboratories scored between 90 and 95 (n = 26), and all other laboratories achieved a score of 100. Thirteen failed the PT on their first attempt, resulting in in-depth troubleshooting to identify root causes for errors and remediation (Table 4). Failing laboratories were issued a second PT panel for repeat testing, eight labs passed, and one lab failed again. One laboratory was not issued a repeat PT panel because remediation was not completed until after the season was closed. Regional differences were noted but not significant, with AFR, AMR, and SEAR regions showing the best results, with all labs passing (one SEAR lab did not participate in 2021 and 2022).

In 2022, PT panels were assessed for 127 laboratories (129 labs invited) with passing scores for 118 (91.5%), and only 9 labs failed the PT panel the first time (Table 3). After the remediation, seven labs passed their second attempt. Five new labs participated for the first time in the ITD PT panel and were invited to submit their results after passing the practice panel with scores >90. Overall, most labs were highly proficient in passing the mEQA on their first attempt. There were 52 laboratories that submitted their results using the online web interface (GPLNMS), and after review of run files, the mEQA results were uploaded to the website by the CDC Polio Team, and an automated email informed the lab of the scores. Remediation was either performed by issuing a new PT panel to the lab or a virtual training using virtual PT panels (raw data files for interpretation), followed by issuance of another PT panel. In both years, only two labs were unable to repeat the panel, or communication dropped. The number of participating labs in all regions remained steady except for the EUR region, which added 14 new labs between the 2020 and 2022 seasons.

### 3.2. Variability in Laboratories

In both years, any lab that requested the PT panel was ready to perform and report the results. Very few labs asked for a delayed delivery due to National Holiday(s) or shortages in staffing due to the conflicting diagnostic testing due to the SARS-Co-V 2 pandemic. The breakdown per error category showed that about 9% and 14% in 2021 and 2022, respectively, made reporting mistakes (incorrect poliovirus serotype or missing mixtures). For 14% and 12% of submitting labs, a combination of technical errors was observed, resulting from interpretation errors (incorrectly called a negative result positive or failed PCR controls) or due to an incorrect assay setup (such as wrong dye or no target selected), or due to an incorrect PCR run method (Table 4). Timeliness issues were observed in less than 3% of the participating labs in either year. Few labs received deductions for failure to follow the correct testing algorithm or mistakes in reporting, with little variation in 2021 and 2022 (5.8% and 6.5%). Examples included not performing the reflex assays for mixtures of Sabin-like 1 or 3-positive RNAs for any mixtures of Sabin and other poliovirus types (Table 4). Most labs were capable of reporting results correctly, but a slight increase was observed in misreporting from 9% in 2021 to 13.8% in 2022. Inversely, labs improved on assay setup in 2022 with only 13 observations instead of 26 the year prior. The most challenging RNA transcripts were samples 12 and 13, with nine labs incorrectly reporting.

Most laboratories submitted their results that were generated using AB 7500 (67%) or CFX96 cyclers (Bio-Rad, 17%), followed by Rotor Gene Q (Qiagen) and Quant Studio cyclers (Thermo Fisher Scientific). Only a few laboratories used other cyclers that had not been validated by the CDC Polio Branch, and assessments were made using either screenshots of the results (Quant Studio 12, Thermo Fisher) or specific PCR cycler software to evaluate runs (Design and Analysis version 2.6.0 software, Quant Studio 7, Thermo Fisher).

## 4. Discussion

The annual molecular proficiency testing for GPLN labs is part of the accreditation process in the worldwide network and has been a pillar for quality assessments and quality assurance since its inception in 1989. This study reports the results of the years 2021 and 2022 of the mEQA for poliovirus combined ITD and VDPV testing panel for the WHO GPLN laboratories in all six WHO regions. The scheme follows GPLN methods and the poliovirus testing and typing algorithm using a suite of PCR assays, followed by reflex assays for referrals. Performance within the GPLN was high, with 86% of labs scoring a passing score of 90 or greater in 2021 (in their first attempt), and this increased to 90% in 2022. Most inaccuracies were due to technical errors such as curve interpretation, assay setup or run method setup, as well as reporting errors. Several labs in the WHO European Region that have participated for the first time in the mEQA PT panel were able to pass at their first attempt, showing that the design and level of complexity can be overcome by initially assessing the lab’s capability on a practice PT panel.

Remediation was initiated by regional polio laboratory coordinators and entailed a data review, repeating another PT panel, or a combination of a deeper root cause analysis performed by the laboratory, followed by virtual PT panels reviewed by the CDC Polio Team. After remediation failed for the second time, the labs were encouraged to attend training in their region to mitigate the performance issues.

Several labs used the online submission tool in GPLNMS in 2022 (n = 52), which provided a smooth experience through reporting with drop-down menus (decreasing the chance of entering an error due to incorrect terminology). The communication was streamlined through the website with automatic emails to submitters, and the PT panel grading was facilitated through concise data collection and real-time reporting. Labs uploaded all relevant PCR run files and submitted metadata, such as the lot number of PCR kits and PCR cyclers, which enabled a quicker analysis of the 2022 PT panel progress.

The PCR assays do not have any CT value cut-off, making interpretation potentially more difficult for inexperienced labs not accustomed to the assay’s curves (sigmoidal curves), hence multiple errors in curve interpretation were observed, but these dropped sharply from 2021 to 2022. The PT panel composition did not vary significantly between the two years; the only difference was the randomization of each version. Due to different PCR cyclers being used in the network and the different assay sensitivities, the CT values for each of the Unknown RNA transcripts were assessed using the most common cyclers (ABI 7500 and CFX96). This is a limitation for the mEQA and previous attempts to decrease concentration for RNA transcripts failed, due to the low sensitivity of some assays that could not consistently detect the RNA target at a very low concentration (such as the least sensitive assays for WPV1, and VDPV3 targets in the ITD suite) [8,9]. Some errors that were observed in both years are largely avoidable through careful supervision by senior scientists (required since 2023). For example, the assay protocols provided inside the kit (each ITD and VDPV kit insert comes folded in the box) detail the run method and the setup of fluorescent dyes for each cycler.

Most laboratories were already IRR registrants and able to order and receive poliovirus testing reagents including PT panels. Several laboratories needed frequent email reminders and engagement by the regional coordinator before PT panels were completed in both years. Despite the RNA being non-infectious transcripts, some countries required extensive import documentation, hindering the shipment within the expected timeframe for the PT panel receipt and completion. Annual mEQA PT panels are typically released between September and November for GPLN; this can lead to issues due to national holidays. In a network of this size (n = 144 labs), it is impossible to align the release and completion for the 6 WHO regions for all PT panels (including the tiered PT panels for virus isolation or sequencing).

For future PT panels, more realistic RNA concentrations need to be assessed to prepare labs for future testing algorithms, for example, using RNA extracted from stools. One RNA transcript sample contained Qβ bacteriophage in preparation for an anticipated change in the GPLN methodology from virus isolation to RNA extraction from AFP specimens and PCR testing. In the future, Qβ will be used to monitor the RNA extraction success and assist in checking for inconsistencies due to the method’s performance [13]. The Qβ target has been helpful in assessing the presence of inhibitors due to inefficient extraction. In 2023, all PT panels were reported through GPLNMS, streamlining the process of reporting results for the grading lab and the submitters. Despite the SARS-CoV-2 pandemic, the GPLN maintained its proficiency while handling an increased need for respiratory specimen testing in the National Labs during the COVID-19 pandemic.

## 5. Conclusions

Most laboratories participating in the GPLN maintained their proficiency in poliovirus molecular detection during the COVID-19 pandemic in 2021 and 2022. GPLN laboratories consistently performed at a high level overall, and assay updates were implemented with proper adherence to protocols and package inserts. Molecular EQA PT panel testing provides WHO and the Global Polio Laboratory Network with valuable data to understand testing capabilities as well as identify training and technical support needs.

## Figures and Tables

**Table 1:** Characteristics of the RNA transcripts used in mEQA for polio ITD and VDPV screening in the WHO GPLN, 2021–2022.

Sample ID	Composition (RNA Transcripts)	Serotype/Genotype PCR Target	Expected Result in ITD/VDPV Algorithm
1	WPV1, Sabin 1 or WPV1, Sabin 3	WPV1, Sabin 1 or WPV1, Sabin 3	NSL1 + SL1 or NSL1 + SL3
2	VDPV1 or VDPV3	VDPV1, or VDPV3	SL3 or SL1 discordant
3	Sabin 3	Sabin 3	SL3
4	Sabin 1 (low concentration), Sabin 3 (high)	Sabin 1, Sabin 3	SL1 + SL3
5	WPV1 or Sabin 1	EV 5′UTR, WPV1, or EV 5′UTR, Sabin 1	Invalid NSL1, SL1
6	WPV1, Sabin 1, Sabin 3	WPV1, Sabin 1, Sabin 3	NSL1 + SL1 + SL3
7	WPV3	WPV3	NSL3
8	Sabin 1, VPDV3	Sabin 1, VDPV3	SL1 + SL3 discordant
9	Sabin 2 or nOPV2	Sabin 2 or nOPV2	PV2
10	nOPV2, WPV1	nOPV2, WPV1	PV2 + NSL1
11	EV	EV 5′UTR	NPEV, non-polio-enterovirus
12	EV, PV	EV 5′UTR, PanPV	Indeterminate
13	Water, Qβ only	Qβ	Non-enterovirus (Qβ positive)

Note: discordant: vaccine-derived poliovirus, EV: enterovirus, invalid: PCR invalid (negative in PanPV or EV target), indeterminate: no serotype positive (PanPV and EV detected), NEV: non-enterovirus (negative), NSL: non-Sabin-like, Qβ: Qβ bacteriophage (non-poliovirus target for extraction control), PanPV: assay to detect any poliovirus, EV-5′UTR: 5′ untranslated region in enterovirus genome (including poliovirus).

**Table 2:** Scoring scheme for PT panel in 2021 and 2022. Abbreviations: PV2-poliovirus type 2, Qβ-bacteriophage, VDPV-vaccine-derived-poliovirus, WPV-wildtype poliovirus.

Scoring Scheme	Deduction	Comments
Major deductions for incorrect results	−15	Failure to detect/identify WPV, VDPV, PV2, or indeterminate
	−10	Failure to detect a single Sabin virus or invalid
	−5	Failure to detect a Sabin virus in a mixture
Deductions for technical issues	−10	Not recognizing failed control(s)
	−5	Failure to follow algorithm or correctly reporting results
	−5	Incorrectly interpreting curves (e.g., entering “positive” on a negative result)
	−5	Failure to correctly set up and report Qβ target in WPV1/Qβ duplex
Late reporting	−5	Per week for any results received > 7 days after panel receipt
Readiness	−15	Not ready to process panel with available reagents or personnel

**Table 3:** Regional scores for mEQA PT panels in 2021 and 2022 (passing scores 90–100, failing scores < 90) and percentages of overall performances.

	Year 2021				Year 2022			
WHO Region	Labs Invited	Passed	Failed	Not Participated	Labs Invited	Passed	Failed	Not Participated
AFR	16	16	0	0	16	13	3	0
AMR	11	8	0	3	11	8	2	1
EMR	11	9	2	0	11	10	1	0
EUR	27	17	9	1	33	30	3	0
SEAR	16	15	0	1	16	15	0	1
WPR	42	40	2	0	42	42	0	0
TOTAL (%)	123 (100%)	105 (86%)	13 (11%)	5 (4%)	129 (100%)	118 (91.5%)	9 (7%)	2 (1.6%)
After remediation (%)	123	113 (91.9%)	5 (4%)	5 (4%)	129	125 (96.9%)	2 (1.6%)	2 (1.6%)

**Table 4:** Summary of PT panel year 2021 and 2022 by error category. Counts were reported with multiple errors per lab; Only the errors were counted if deductions were made to the overall score.

PT Panel Year		2021		2022	
Number of labs participating (total)		118		127	
Category of errors (any observation if score <100)		Number of observations	Percentage (of total labs)	Number of observations	Percentage (of total labs)
Reporting		11	9.1%	17	13.8%
Timeliness (>7 days)		2	1.7%	3	2.4%
Following algorithm		7	5.8%	8	6.5%
Technical (overall)		17	14.0%	15	12.2%
	Curve interpretation	17	14.0%	5	4.1%
	Assay set up	26	21.5%	13	10.2%
	Run method set up	9	7.4%	5	4.1%

## Data Availability

The datasets presented in this article are not readily available because the data are part of the restricted system supporting the accreditation of the Global Polio Network Laboratories, individual laboratory data will not be disclosed due to GPEI and WHO limitations. Requests to access the datasets should be directed to www.polioeradication.org.
